# Redetermination of 4-cyano­pyridine *N*-oxide

**DOI:** 10.1107/S1600536812036690

**Published:** 2012-08-31

**Authors:** Rodolfo Moreno-Fuquen, Carolina Arana, Carlos A. De Simone

**Affiliations:** a Departamento de Química - Facultad de Ciencias, Universidad del Valle, Apartado 25360, Santiago de Cali, Colombia; b Instituto de Física de São Carlos, IFSC, Universidade de São Paulo, USP, São Carlos, SP, Brazil

## Abstract

In the title pyridine *N*-oxide derivative, C_6_H_4_N_2_O, the 4-cyano substituent almost lies in the mean plane of the pyridine ring (r.m.s deviation of all non-H atoms = 0.004 Å). This redetermination results in a crystal structure with significantly higher precision [N—O bond length is 1.2997 (15) compared with 1.303 (5) Å in the original] than the original determination, which was recorded using the multiple-film technique and visually estimated intensities [Hardcastle *et al.* (1974 ▶). *J. Cryst. Mol. Struct.* **4**, 305–311]. The crystal structure features weak C—H⋯O and C—H⋯N inter­actions, which lead to the formation of chains that inter­sect each other parallel to (001).

## Related literature

For the synthesis of 4-cyano­pyridine *N*-oxide with metal ions, see: Piovesana & Selbin (1969 ▶). For luminiscent properties of 4-cyano­pyridine *N*-oxide lanthanide complexes, see: Eliseeva *et al.* (2006 ▶, 2008 ▶). For the use of the title compound as a ligand to obtain metal-organic coordination polymers, see: Yang *et al.* (2009 ▶); Kapoor *et al.* (2012 ▶). For details concerning thermodynamic studies of the title compound, see: Ribeiro *et al.* (1998 ▶). For hydrogen bonding, see: Nardelli (1995 ▶). For the previous determination of the structure, see: Hardcastle *et al.* (1974 ▶).
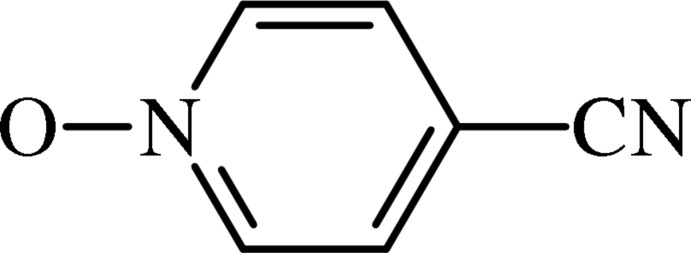


## Experimental

### 

#### Crystal data

C_6_H_4_N_2_O
*M* *_r_* = 120.11
Monoclinic, 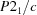

*a* = 7.8743 (8) Å
*b* = 6.0582 (6) Å
*c* = 11.6278 (10) Å
β = 91.973 (6)°
*V* = 554.36 (9) Å^3^
*Z* = 4
Mo *K*α radiation
μ = 0.10 mm^−1^
*T* = 295 K
0.37 × 0.32 × 0.30 mm

#### Data collection

Nonius KappaCCD diffractometer
4336 measured reflections
1224 independent reflections
964 reflections with *I* > 2σ(*I*)
*R*_int_ = 0.037

#### Refinement

*R*[*F* ^2^ > 2σ(*F* ^2^)] = 0.057
*wR*(*F* ^2^) = 0.169
*S* = 1.10
1224 reflections
82 parameters
H-atom parameters constrained
Δρ_max_ = 0.25 e Å^−3^
Δρ_min_ = −0.21 e Å^−3^

### 

Data collection: *COLLECT* (Nonius, 2000 ▶); cell refinement: *SCALEPACK* (Otwinowski & Minor, 1997 ▶); data reduction: *DENZO* (Otwinowski & Minor, 1997 ▶) and *SCALEPACK*; program(s) used to solve structure: *SHELXS97* (Sheldrick, 2008 ▶); program(s) used to refine structure: *SHELXL97* (Sheldrick, 2008 ▶); molecular graphics: *ORTEP-3 for Windows* (Farrugia, 1997 ▶) and *Mercury* (Macrae *et al.*, 2006 ▶); software used to prepare material for publication: *WinGX* (Farrugia, 1999 ▶).

## Supplementary Material

Crystal structure: contains datablock(s) I, global. DOI: 10.1107/S1600536812036690/hg5244sup1.cif

Structure factors: contains datablock(s) I. DOI: 10.1107/S1600536812036690/hg5244Isup2.hkl

Supplementary material file. DOI: 10.1107/S1600536812036690/hg5244Isup3.cml

Additional supplementary materials:  crystallographic information; 3D view; checkCIF report

## Figures and Tables

**Table 1:** Hydrogen-bond geometry (Å, °)

*D*—H⋯*A*	*D*—H	H⋯*A*	*D*⋯*A*	*D*—H⋯*A*
C1—H1⋯O1^i^	0.93	2.35	3.200 (2)	152
C5—H5⋯O1^ii^	0.93	2.43	3.323 (2)	161
C2—H2⋯N2^iii^	0.93	2.68	3.530 (2)	153

Symmetry codes: (i) 
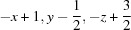
; (ii) 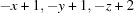; (iii) 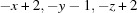.

**Fig. 1.**
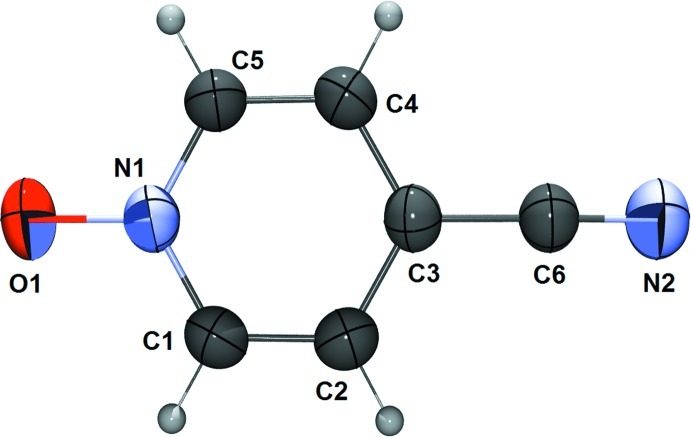
Molecular conformation and atom numbering scheme for the title compound with displacement ellipsoids drawn at the 50% probability level. H atoms are shown as spheres of arbitrary radius.

**Fig. 2.**
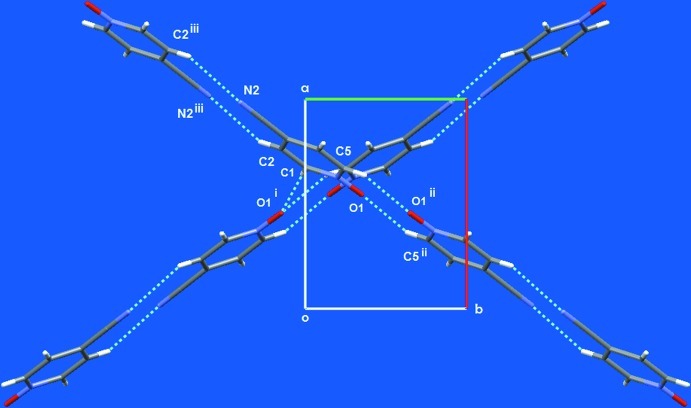
Part of the crystal structure of (I), showing the formation of chains which running parallel to (001). Symmetry code: (i) -x+1,+y-1/2,-z+3/2; (ii) -x+1,-y+1,-z+2; (iii) -x+2,-y-1,-z+2.

## supplementary crystallographic information

### Comment

The incorporation of 4-cyanopyridine N-oxide, (I) as ligand in the formation of diverse complexes with metal ions has been known for a long time (Piovesana & Selbin, 1969). This compound has also been used in the formation of dimeric lanthanide complexes showing luminiscent properties (Eliseeva *et al.*, 2006 and Eliseeva *et al.*, 2008). A series of metal-organic supramolecular co-ordinated polymers with the 4-cyanopyridine N-oxide as a ligand was also reported (Yang *et al.*, 2009; Kapoor *et al.*, 2012). Thermodynamic studies of diverse N-oxide components, including (I) compound, have also been reported (Ribeiro *et al.*, 1998). As part of our studies on the substituent effects on the structures it was necessary to know the structural behavior of the 4-cyanopyridine N-oxide. The crystal and molecular structure of (I) had been determined before, but with low precision. Thus, the redetermination of the title compound (Fig. 1), results in a crystal structure with significantly higher precision than the original determination which was recorded using the multiple-film technique and visually estimated intensities (Hardcastle *et al.*, (1974). Obtaining a more orthogonal cell compared with the original analysis, allows a more precise picture of the packing in the crystal structure. The pyridine ring is essentially planar (r.m.s. deviation of all non-hydrogen atoms = 0.004 Å) The plane formed by N2-C6-C3 atoms, which is part of the cyano group forms an angle of 2.7 (1) Å with the plane of pyridine. The pyridine ring bond lengths and bond angles of (I) are normal and are close to the values presented earlier for this same structure (Hardcastle *et al.*, (1974). In the crystal, there are no classical hydrogen bonds. The crystal structure is stabilized by intermolecular C—H···O and C—H···N weak interactions, which lead to the formation of chains of molecules that intersect each other parallel to (001), (Table 1 and Fig. 2). Indeed, the chains of molecules are formed by weak C5—H5···O1 and C2—H2···N2 interactions (Nardelli, 1995). In turn, these chains are linked by C1—H1···O1 interactions.

### Experimental

Commercial 4-cyanopyridine N-oxide [CAS-14906-59-3] (Aldrich) was recrystallized from acetonitrile.

### Refinement

All H-atoms were positioned at geometrically idealized positions with C—H distance of 0.93 Å and U_iso_(H) = 1.2 times U_eq_ of the C-atoms to which they were bonded.

### Crystal data

**Table:**

C_6_H_4_N_2_O	*F*(000) = 248
*M**_r_* = 120.11	*D*_x_ = 1.439 Mg m^−^^3^
Monoclinic, *P*2_1_/*c*	Melting point: 496(1) K
Hall symbol: -P 2ybc	Mo *K*α radiation, λ = 0.71073 Å
*a* = 7.8743 (8) Å	Cell parameters from 7553 reflections
*b* = 6.0582 (6) Å	θ = 2.6–27.5°
*c* = 11.6278 (10) Å	µ = 0.10 mm^−^^1^
β = 91.973 (6)°	*T* = 295 K
*V* = 554.36 (9) Å^3^	Block, pale-green
*Z* = 4	0.37 × 0.32 × 0.30 mm

### Data collection

**Table:**

Nonius KappaCCD diffractometer	964 reflections with *I* > 2σ(*I*)
Radiation source: fine-focus sealed tube	*R*_int_ = 0.037
Graphite monochromator	θ_max_ = 27.6°, θ_min_ = 2.6°
CCD rotation images, thick slices scans	*h* = −9→10
4336 measured reflections	*k* = −7→7
1224 independent reflections	*l* = −15→14

### Refinement

**Table:**

Refinement on *F*^2^	Primary atom site location: structure-invariant direct methods
Least-squares matrix: full	Secondary atom site location: difference Fourier map
*R*[*F*^2^ > 2σ(*F*^2^)] = 0.057	Hydrogen site location: inferred from neighbouring sites
*wR*(*F*^2^) = 0.169	H-atom parameters constrained
*S* = 1.10	*w* = 1/[σ^2^(*F*_o_^2^) + (0.1003*P*)^2^ + 0.0643*P*] where *P* = (*F*_o_^2^ + 2*F*_c_^2^)/3
1224 reflections	(Δ/σ)_max_ < 0.001
82 parameters	Δρ_max_ = 0.25 e Å^−^^3^
0 restraints	Δρ_min_ = −0.21 e Å^−^^3^

### Special details

**Table:**

Geometry. All esds (except the esd in the dihedral angle between two l.s. planes) are estimated using the full covariance matrix. The cell esds are taken into account individually in the estimation of esds in distances, angles and torsion angles; correlations between esds in cell parameters are only used when they are defined by crystal symmetry. An approximate (isotropic) treatment of cell esds is used for estimating esds involving l.s. planes.
Refinement. Refinement of *F*^2^ against ALL reflections. The weighted *R*-factor *wR* and goodness of fit *S* are based on *F*^2^, conventional *R*-factors *R* are based on *F*, with *F* set to zero for negative *F*^2^. The threshold expression of *F*^2^ > σ(*F*^2^) is used only for calculating *R*-factors(gt) *etc*. and is not relevant to the choice of reflections for refinement. *R*-factors based on *F*^2^ are statistically about twice as large as those based on *F*, and *R*- factors based on ALL data will be even larger.

### Fractional atomic coordinates and isotropic or equivalent isotropic displacement parameters (Å^2^)

**Table:**

	*x*	*y*	*z*	*U*_iso_*/*U*_eq_	
N1	0.62819 (15)	0.2012 (2)	0.91581 (10)	0.0456 (4)	
O1	0.54449 (17)	0.3499 (2)	0.85653 (10)	0.0671 (5)	
C4	0.7599 (2)	0.0848 (3)	1.09136 (14)	0.0500 (4)	
H4	0.7885	0.1123	1.1683	0.060*	
C6	0.90575 (19)	−0.2718 (2)	1.10517 (13)	0.0482 (4)	
N2	0.9834 (2)	−0.4015 (2)	1.15568 (14)	0.0656 (5)	
C3	0.80898 (17)	−0.1108 (2)	1.04066 (12)	0.0427 (4)	
C2	0.7643 (2)	−0.1471 (3)	0.92614 (13)	0.0500 (4)	
H2	0.7958	−0.2775	0.8905	0.060*	
C5	0.6695 (2)	0.2377 (3)	1.02802 (13)	0.0504 (4)	
H5	0.6360	0.3683	1.0626	0.061*	
C1	0.6735 (2)	0.0094 (2)	0.86553 (13)	0.0523 (5)	
H1	0.6426	−0.0162	0.7888	0.063*	

### Atomic displacement parameters (Å^2^)

**Table:**

	*U*^11^	*U*^22^	*U*^33^	*U*^12^	*U*^13^	*U*^23^
N1	0.0492 (7)	0.0422 (7)	0.0449 (7)	0.0025 (5)	−0.0066 (5)	0.0031 (5)
O1	0.0830 (9)	0.0573 (8)	0.0596 (7)	0.0187 (6)	−0.0172 (6)	0.0103 (6)
C4	0.0580 (9)	0.0493 (9)	0.0422 (8)	0.0037 (7)	−0.0078 (7)	−0.0040 (6)
C6	0.0508 (8)	0.0445 (8)	0.0489 (8)	0.0025 (6)	−0.0047 (7)	0.0019 (7)
N2	0.0726 (10)	0.0572 (9)	0.0660 (10)	0.0104 (7)	−0.0116 (7)	0.0040 (7)
C3	0.0415 (7)	0.0410 (8)	0.0454 (8)	−0.0015 (5)	−0.0019 (6)	0.0038 (6)
C2	0.0576 (9)	0.0429 (8)	0.0491 (9)	0.0032 (6)	−0.0029 (7)	−0.0055 (6)
C5	0.0607 (9)	0.0435 (8)	0.0465 (8)	0.0057 (7)	−0.0071 (7)	−0.0057 (6)
C1	0.0643 (10)	0.0504 (9)	0.0417 (8)	0.0030 (7)	−0.0079 (7)	−0.0044 (7)

### Geometric parameters (Å, º)

**Table:**

N1—O1	1.2997 (15)	C6—C3	1.4336 (19)
N1—C5	1.3520 (19)	C3—C2	1.383 (2)
N1—C1	1.354 (2)	C2—C1	1.368 (2)
C4—C5	1.368 (2)	C2—H2	0.9300
C4—C3	1.385 (2)	C5—H5	0.9300
C4—H4	0.9300	C1—H1	0.9300
C6—N2	1.1453 (19)		
			
O1—N1—C5	119.96 (13)	C1—C2—C3	119.79 (14)
O1—N1—C1	120.14 (13)	C1—C2—H2	120.1
C5—N1—C1	119.90 (13)	C3—C2—H2	120.1
C5—C4—C3	119.86 (14)	N1—C5—C4	120.84 (14)
C5—C4—H4	120.1	N1—C5—H5	119.6
C3—C4—H4	120.1	C4—C5—H5	119.6
N2—C6—C3	179.31 (16)	N1—C1—C2	120.87 (14)
C2—C3—C4	118.71 (14)	N1—C1—H1	119.6
C2—C3—C6	120.58 (13)	C2—C1—H1	119.6
C4—C3—C6	120.71 (13)		
			
C5—C4—C3—C2	−0.2 (2)	C1—N1—C5—C4	1.4 (2)
C5—C4—C3—C6	179.17 (14)	C3—C4—C5—N1	−0.5 (3)
N2—C6—C3—C4	−167 (14)	O1—N1—C1—C2	178.83 (15)
C4—C3—C2—C1	0.1 (2)	C5—N1—C1—C2	−1.4 (2)
C6—C3—C2—C1	−179.26 (14)	C3—C2—C1—N1	0.7 (2)
O1—N1—C5—C4	−178.92 (14)		

### Hydrogen-bond geometry (Å, º)

**Table:**

*D*—H···*A*	*D*—H	H···*A*	*D*···*A*	*D*—H···*A*
C1—H1···O1^i^	0.93	2.35	3.200 (2)	152
C5—H5···O1^ii^	0.93	2.43	3.323 (2)	161
C2—H2···N2^iii^	0.93	2.68	3.530 (2)	153

Symmetry codes: (i) −*x*+1, *y*−1/2, −*z*+3/2; (ii) −*x*+1, −*y*+1, −*z*+2; (iii) −*x*+2, −*y*−1, −*z*+2.
